# Facile Bottom-up Preparation of WS_2_-Based Water-Soluble Quantum Dots as Luminescent Probes for Hydrogen Peroxide and Glucose

**DOI:** 10.1186/s11671-019-3109-5

**Published:** 2019-08-09

**Authors:** Da-Ren Hang, De-You Sun, Chun-Hu Chen, Hui-Fen Wu, Mitch M. C. Chou, Sk Emdadul Islam, Krishna Hari Sharma

**Affiliations:** 1 Department of Materials and Optoelectronic Science, National Sun Yat-sen University, Kaohsiung, 80424 Taiwan; 2 Center of Crystal Research, National Sun Yat-sen University, Kaohsiung, 80424 Taiwan; 3 Department of Chemistry, National Sun Yat-sen University, Kaohsiung, 80424 Taiwan

**Keywords:** Semiconductors, Quantum localization, Chemical synthesis, Luminescence, Optical properties

## Abstract

Photoluminescent zero-dimensional (0D) quantum dots (QDs) derived from transition metal dichalcogenides, particularly molybdenum disulfide, are presently in the spotlight for their advantageous characteristics for optoelectronics, imaging, and sensors. Nevertheless, up to now, little work has been done to synthesize and explore photoluminescent 0D WS_2_ QDs, especially by a bottom-up strategy without using usual toxic organic solvents. In this work, we report a facile bottom-up strategy to synthesize high-quality water-soluble tungsten disulfide (WS_2_) QDs through hydrothermal reaction by using sodium tungstate dihydrate and l-cysteine as W and S sources. Besides, hybrid carbon quantum dots/WS_2_ QDs were further prepared based on this method. Physicochemical and structural analysis of QD hybrid indicated that the graphitic carbon quantum dots with diameters about 5 nm were held onto WS_2_ QDs via electrostatic attraction forces. The resultant QDs show good water solubility and stable photoluminescence (PL). The excitation-dependent PL can be attributed to the polydispersity of the synthesized QDs. We found that the PL was stable under continuous irradiation of UV light but can be quenched in the presence of hydrogen peroxide (H_2_O_2_). The obtained WS_2_-based QDs were thus adopted as an electrodeless luminescent probe for H_2_O_2_ and for enzymatic sensing of glucose. The hybrid QDs were shown to have a more sensitive LOD in the case of glucose sensing. The Raman study implied that H_2_O_2_ causes the partial oxidation of QDs, which may lead to oxidation-induced quenching. Overall, the presented strategy provides a general guideline for facile and low-cost synthesis of other water-soluble layered material QDs and relevant hybrids in large quantity. These WS_2_-based high-quality water-soluble QDs should be promising for a wide range of applications in optoelectronics, environmental monitoring, medical imaging, and photocatalysis.

**Electronic supplementary material:**

The online version of this article (10.1186/s11671-019-3109-5) contains supplementary material, which is available to authorized users.

## Introduction

In the past decade, graphene has opened a new horizon of two-dimensional (2D) materials for chemists and physicists [1–3]. Due to the inherent shortcomings of graphene, such as absence of band gap, research for other kinds of 2D materials is currently in the spotlight. Notable 2D material groups include layered transition metal dichalcogenides (TMDs), layered transition metal oxides, and carbide-based materials [4–8]. The characteristic 2D structure of TMD results in anisotropic physical properties, ranging from electron mobility to catalytic and optical properties. In comparison with their bulk counterpart, the general advantages of ultra-thin TMDs are the tunable physical properties and the enriched active sites for chemical reactions. As the most popular 2D TMD material, single-layer or multilayer molybdenum disulfide (MoS_2_) has shown great potential in a wide range of applications, such as electronics, sensors, and photocatalysis [9–11]. Especially, ultrathin atomic-layered MoS_2_ holds great promise for constructing biosensors because high specific surface area and ample active surface states make 2D MoS_2_ very sensitive to exposure to target analytes. In the field of biosensing, 2D MoS_2_ has a relatively low toxicity in comparison to many other nanomaterials, in particular, graphene and graphene oxides [12]. For instance, 2D MoS_2_ has been employed for the detection of hydrogen peroxide (H_2_O_2_) and glucose in the last couple of years [13–15].

The detection of hydrogen peroxide, a vital reactive oxygen species, is of practical importance in chemical, pharmaceutical, clinical, and environmental fields. For example, an abnormal high level of H_2_O_2_ could mean the generation of acid rain and could indicate the risk of a few diseases like Alzheimer’s disease and Parkinson’s disease [16]. On the other hand, glucose plays an important role in biochemical pathway and human health evaluation. Convenient and cheap detection of glucose is of considerable significance in the diabetes mellitus diagnosis, food, and biofuel cell analysis. Besides, it is known that over 80% of biosensor industry research is related to glucose sensors. Therefore, the development of a facile, low-priced, and accurate sensor for H_2_O_2_ and glucose continue to receive tremendous research effort [17, 18].

Zero-dimensional (0D) quantum dots (QDs) derived from ultrathin 2D materials are emerging as a novel category of nanoscale 0D materials [19, 20]. Compared with TMD nanosheets, TMD QDs show distinct and exceptional physical properties due to pronounced quantum confinement and edge effects. By reducing the dimensions of QDs close to the excitonic Bohr radius, it was found that the quantum confinement effect (QCE) enhanced the photoluminescence (PL) quantum efficiency of MoS_2_ QDs [21, 22]. Moreover, the ultrathin sizes of MoS_2_ QDs lead to larger surface-to-volume ratio and abundant active edge states, making them chemically sensitive to the surroundings. Thus TMD QDs can be promising for use in sensing, luminescence, bioimaging, and catalysis. In this regard, MoS_2_ QDs were lately employed for PL sensor to detect chemical and bioanalyte [23, 24].

Following the successful development of MoS_2_ in various applications, tungsten disulfide (WS_2_) begins to receive increasing amount of attention [25]. The layer structure consists of 2D monolayer building blocks held by weak van der Waals interaction. Each WS_2_ single layer possesses a hexagonal crystal structure formed by covalently bonded S-W-S monolayers, where a tungsten atom sheet is sandwiched by two layers of S atoms. Compared with molybdenum, tungsten has several benefits such as copious natural resources, cheaper prices, and less toxicity, which is favorable for industrial applications. Additionally, the larger size of W provides more spacious interlayer channels in the 2D structure and facilitates physical property modulation via substitutional doping. WS_2_ is also preferential in tungsten dichalcogenides when a high chemical reactivity is in need at the unsaturated sulfur edges. 2D WS_2_ nanosheets have recently found a number of applications, such as FETs [26], photodetectors [27, 28], and photocatalysis [29, 30]. WS_2_ in its bulk form has an indirect bandgap and a photoluminescence (PL) band in infrared with low quantum efficiency [25]. In QD configuration, 0D WS_2_ has a direct bandgap and hence shows highly efficient PL, facilitating the construction of electrodeless optical sensing templates. The resultant PL that appears in the visible range is compatible with most low-cost commercial optical platforms. Advantageously, the noncontact nature of optical sensing supports the future realization of advanced integrated multifunctional microchips.

To date, considerable efforts have been dedicated to achieve the synthesis of photoluminescent MoS_2_ QD materials [22, 31]. In contrast, the progress in the synthesis and application of photoluminescent WS_2_ QDs is still rather limited. In general, synthetic strategies can be divided into “top-down” and “bottom-up” approaches. As for the “top-down” methods, liquid exfoliation methods are usually regarded as an efficient methodology to prepare single or few-layered 2D material suspensions in large quantities. Successful preparations of WS_2_ QDs by intercalation techniques adopting lithium and K ions have been reported [32, 33]. In such cases, hazardous and time-consuming processes were involved. Besides, further purification was required to remove ionic residues and semiconducting properties could be weakened because of ion intercalation. On the other hand, sonication-assisted liquid-phase exfoliation technique is based on high ultrasonic powers and the match of surface tension between the solvents and the targeted stratified bulk materials [34–36]. Several recent reports on the preparation of WS_2_ QDs have employed this rather universal route [37–40]. However, this technique is usually associated with hazardous organic solvents and laborious pretreatment, and is quite sensitive to the environmental conditions. In addition, the derived product is typically plagued with residue solvents. The high-temperature post-treatment process is thus required to get rid of excessive solvents with high boiling points. Nevertheless, it may lead to the aggregation of WS_2_ QDs and the formation of harmful side products in certain cases.

While most of these synthetic routes belong to “top-down” synthesis, the advancement in the “bottom-up” synthesis of photoluminescent WS_2_ QDs is fairly restricted [41, 42]. Among the “bottom-up” chemical synthetic approaches, the hydrothermal method has become a well-received and cost-effective technique for preparing semiconducting nanocrystals. The dimension and morphology of the synthesized nanostructures can be easily controlled by the chemical reaction parameters and precursor selection. In comparison with most “top-down” synthesis, the hydrothermal process is simple, environmentally benign, and well-suited to the facile formation of nanohybrid materials. Moreover, a recent investigation on hydrothermally prepared MoS_2_ QDs suggested that the solubility and stability of MoS_2_ QDs were improved due to some accompanying surface functional groups [24]. Due to these favorable attributes, the exploration of facile hydrothermal synthesis of water-dispersible WS_2_ QDs with stable photoluminescence is significant and urgent at this stage. In this paper, we herein present a facile bottom-up hydrothermal route for the synthesis of photoluminescent WS_2_ QDs. Furthermore, motivated by recent progress in carbon quantum dots (CDs)/2D MoS_2_ composites and to show the viable hybrid formation by hydrothermal protocol, we proceeded to prepare CD/WS_2_ QDs for the first time [43–45]. CDs are 0D quasi-spherical nanoparticles, with diameter in the order of 10 nm or less, showing superb solubility, biocompatibility, photochemical stability, and rapid electron transfer properties [46]. Next, the prepared WS_2_ QDs were characterized in detail. The intense blue emission from synthesized QDs was then used as luminescent probes to construct electrodeless PL sensors for detection of hydrogen peroxide and glucose. Likewise, the sensors displayed a good selectivity toward glucose over other probable interfering species. In the case of glucose sensing, it was found that the hybrid CD/WS_2_ QDs have a more sensitive LOD than that of pristine WS_2_ QDs. The obtained results indicated that the synthesized WS_2_ QDs and novel CD/WS_2_ hybrid QDs possess small sizes, stable and intense PL, high dispersibility, and non-toxicity. We believe that these optical active WS_2_ QDs are promising to serve as new platforms for chemical and biological molecules sensors and other functional devices. Extended studies toward this direction are currently ongoing.

## Methods

### Reagents and Chemicals

Sodium tungstate dihydrate (Na_2_WO_4_·2H_2_O) was obtained from Nihon Shiyaku Reagent (Tokyo, Japan). l-cysteine was purchased from Alfa Aesar. They served as starting materials for the hydrothermal synthesis of WS_2_ QDs. Here, l-cysteine acts as sulfur source as well as reducing agent. Glucose, fructose, maltose, and sucrose were obtained from Honeywell Fluka (Shanghai, China). Lactose, histidine, glycine, potassium chloride, and magnesium chloride were obtained from Sigma-Aldrich. All the reagents were of analytical purity and were used as received without further purification. Throughout the synthesis, ultrapure water from Milli-Q Plus water purification system (Millipore Co., Bedford, MA, USA) was adopted for solution preparation.

### Materials Preparation

#### Synthesis of 0D WS_2_ QDs

**Scheme 1:**
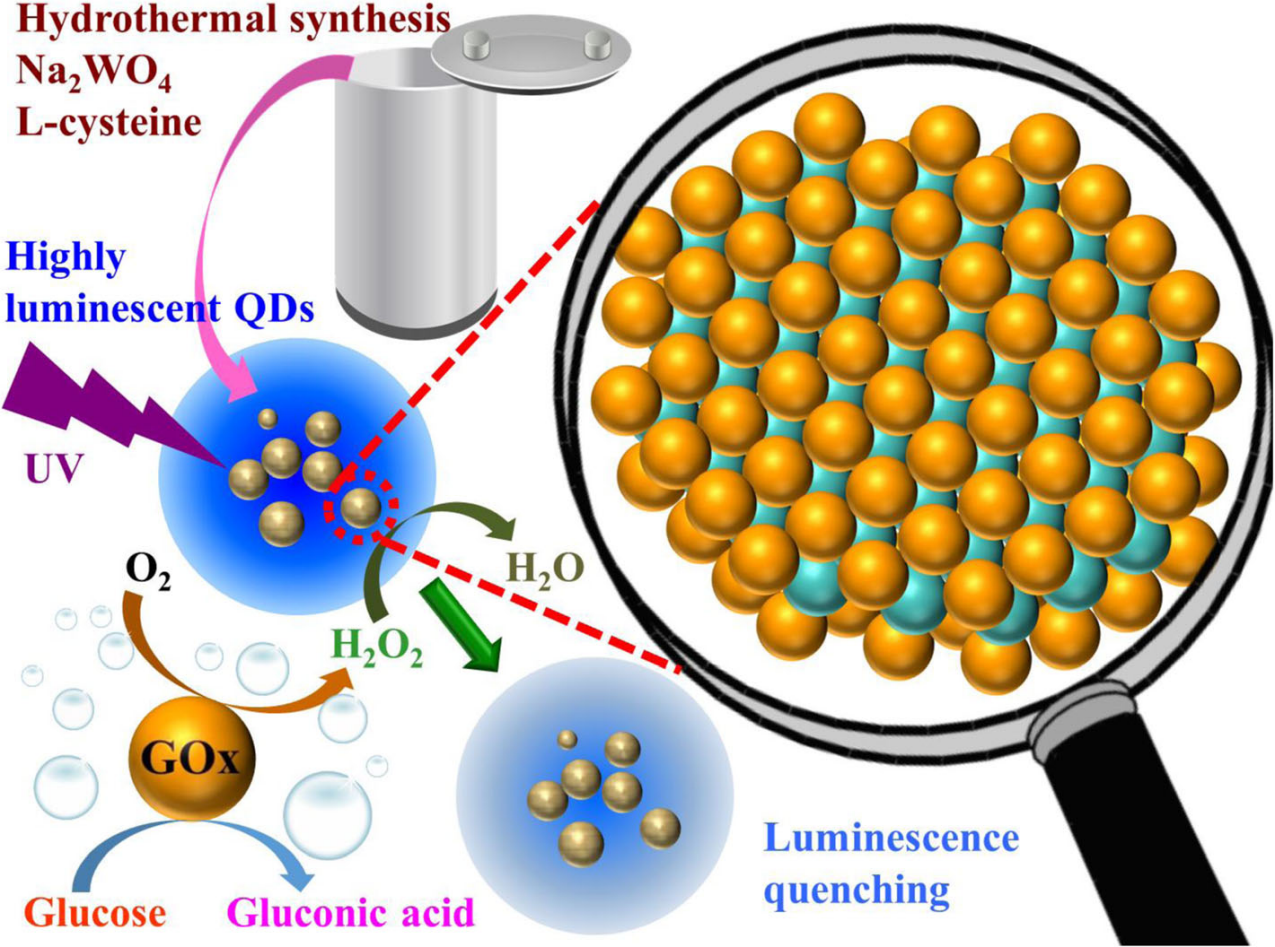
Schematic illustration of detection mechanism for glucose by using WS_2_ QDs. The sensing can be realized via GOx-catalyzed oxidation reaction with dissolved O_2_ in solution. The PL of QDs can be quenched in proportional to the generated H_2_O_2_. (color online)

The water-soluble WS_2_ QDs were synthesized through a facile and one-step hydrothermal method. The synthetic procedure is concisely shown in Scheme 1. In short, 0.066 g of Na_2_WO_4_·2H_2_O was dissolved in 12.5 mL of ultrapure water with further ultrasonication for 5 min. Then 0.1 M HCl was added to adjust the pH to 6.5. Afterward, 0.0242 g of l-cysteine and 50 mL of water were poured into the solution and was followed by ultrasonication for 10 min. The mixture was subsequently transferred into a 100-mL Teflon-lined stainless steel autoclave and reacted at 180 °C for 24 h. After the autoclave cooled naturally, the supernatant containing WS_2_ QDs was centrifuged for 20 min at the speed of 10,000 rpm. The WS_2_ QD product was collected and then stored in a refrigerator at 4 °C.

#### Synthesis of Carbon Quantum Dots

Carbon quantum dots were prepared by an eco-friendly microwave-assisted method, which is analogous to the CD synthesis in previous reports [47, 48]. In a typical production, 17.1 g of sucrose was dissolved in deionized water to prepare 1 M sucrose solution. Next, the solution was subjected to microwave heating at 500 W for 20 min. The CD can be collected and filtered through a filter. After that, the CD solution was stored at 4 °C for further experiments.

#### Synthesis of CD/WS_2_ QDs

For synthesis of hybrid CD/WS_2_ QDs, certain amounts of CD solutions were sonicated for 20 min to achieve uniform dispersion. The CD solution was added to the preceding WS_2_ precursor solution with vigorous stirring for 15 min. Next, the homogeneous mixture was transferred into a 100-mL Teflon-lined autoclave and kept at 180 °C for 24 h. After the suspension was cooled to room temperature, the CD/WS_2_ QDs were collected by using centrifugation for 20 min at 10,000 rpm.

### Material Characterization

The phase structure was characterized by a Siemens D5000 powder diffractometer utilizing Cu_Kα_ radiation (λ = 1.5418 Å). Further microstructural information of the samples was provided by transmission electron microscopy (TEM) and high-resolution transmission electron microscopy (HRTEM) by using a JEOL-3010 transmission electron microscope. X-ray photoelectron spectroscopy (XPS) measurements were carried out with an ultrahigh vacuum JEOL JPS-9010 electron spectrometer equipped with a multi-channel detector. The collected binding energies were referenced to the C1s peaks at 284.6 eV of the surface adventitious carbon. The UV–Vis spectra were recorded with a Jasco V-630 spectrophotometer (USA) with a standard 10-mm path length quartz cuvette. The photoluminescence (PL) and photoluminescence excitation (PLE) spectra of the as-prepared samples were measured using a Hitachi F-4500 florescence spectrophotometer linked to a 150 W Xenon lamp as the excitation source. The PL decay time of the QDs was recorded on an Edinburgh Instruments OB920 Fluorescence Lifetime Spectrometer (Edinburgh Instruments Ltd., Livingston, UK). The Raman measurements were taken in ambient conditions with a red light laser. The scattered light was collected by the same objective lens and dispersed with a Horiba iHR320 spectrometer [49].

## Results and Discussion

### Structural and Morphological Studies

**Fig. 1:**
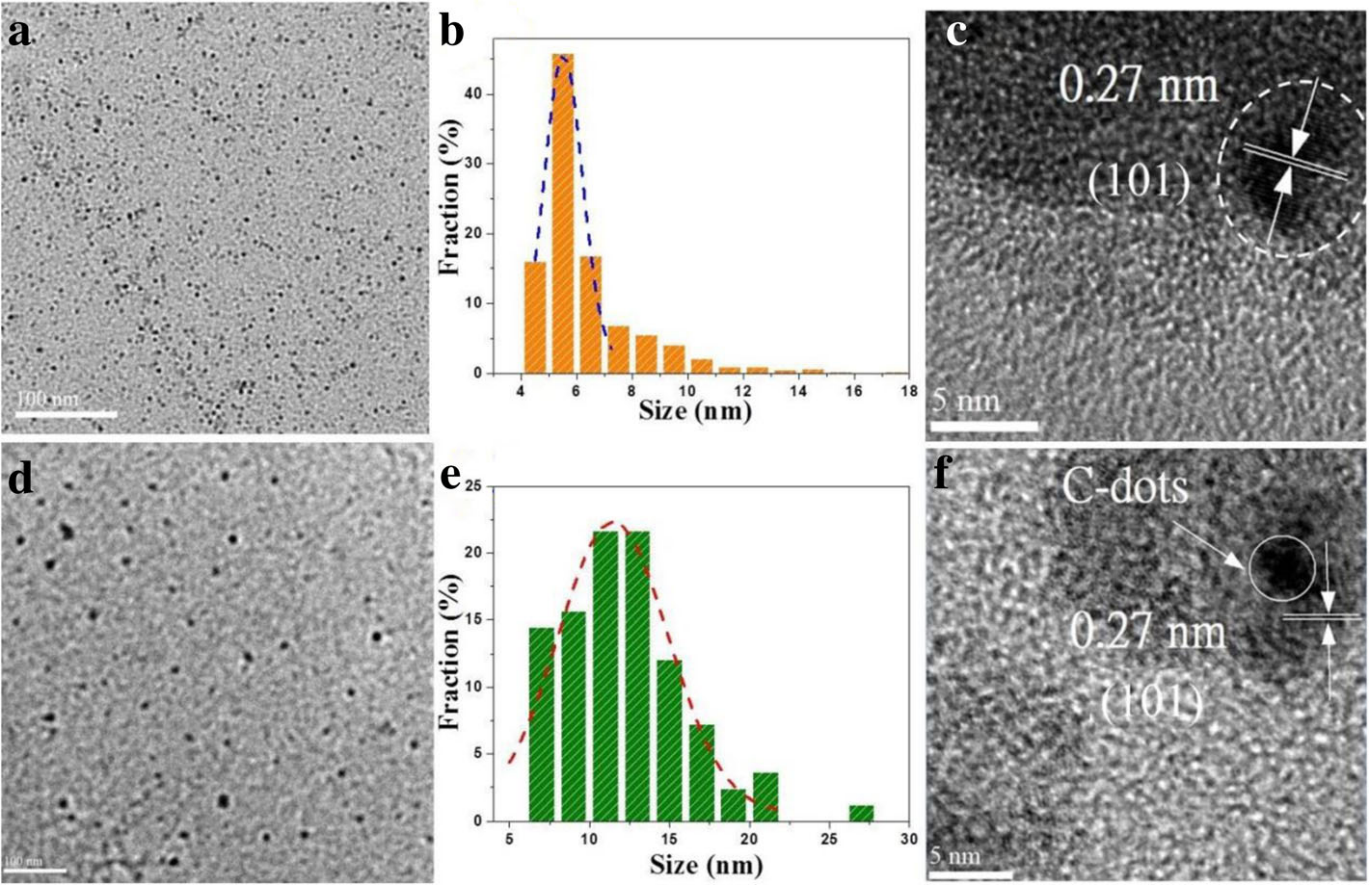
**a** TEM image of WS_2_ QDs. **b** Particle size distribution of WS_2_ QDs. **c** Representative HRTEM image of the WS_2_ QD. **d** TEM image of the CD/WS_2_ QDs. **e** Size distribution of CD/WS_2_ QDs. **f** The HRTEM image of CD/WS_2_ QDs showing the preserved crystallinity. (color online)

The facile one-pot hydrothermal process to prepare water-dispersible WS_2_ QDs is tersely illustrated in Scheme 1. The preparation details are described in the experimental section. The structural information of the as-formed WS_2_ QDs was firstly investigated by transmission electron microscopy (TEM) and high-resolution transmission electron microscopy (HRTEM), as shown in Fig. 1. A typical TEM image of the resultant WS_2_ QDs (Fig. 1a) shows that the QDs are uniformly dispersed in aqueous phase without apparent aggregation. The excellent water solubility can be derived by residual hydrophilic amino or carboxyl groups on the synthesized QD surface. The lateral size distribution of QDs is shown by plotting the histogram in Fig. 1b, where up to 76% QDs are distributed in the narrow range from 4 to 7 nm. The HRTEM image in Fig. 1c reveals that the lattice fringe spacing of the WS_2_ QD was 0.27 nm, which is matched with the (101) plane of hexagonal WS_2_ crystal [37, 50]. Figure 1d shows the TEM image of the as-prepared hybrid CD/WS_2_ QDs with good dispersion. The statistical analysis of particle size distribution was conducted and presented in Fig. 1e. It can be found that the average particle size of hybrid QDs is 11.5 nm and the majority lies in the range of 7–15 nm. Figure 1f presents a typical HRTEM image of one of the hybrid QD in which CDs can be found on the QD surface. In addition, the (101) d-spacing of 2H-WS_2_ was once again observed in the hybrid QD as with the pristine QD material, implying that the good crystalline structure was retained after the hybrid formation.

**Fig. 2:**
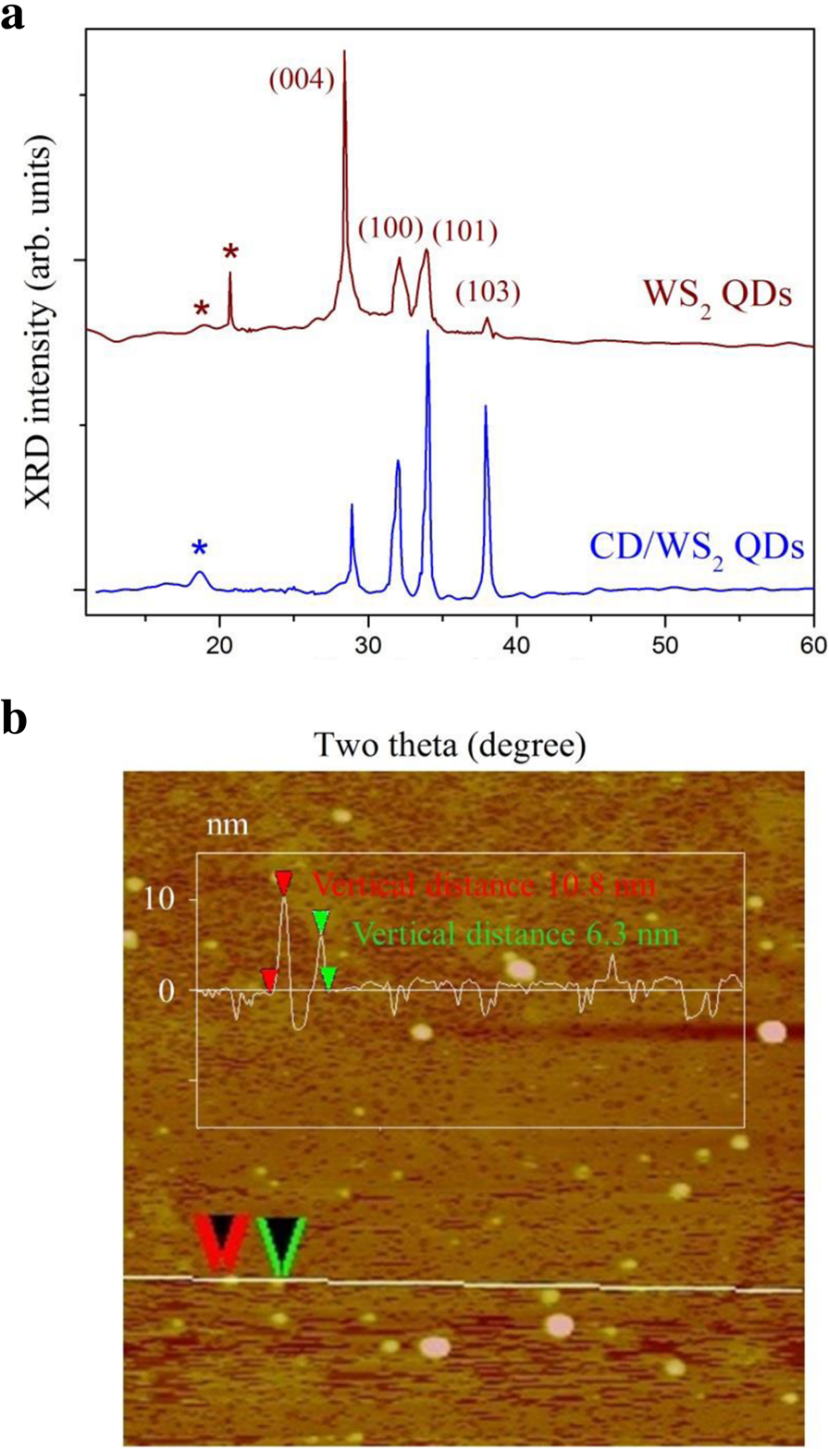
**a** X-ray diffraction patterns of WS_2_ QDs and CD/WS_2_ QD composite. **b** The atomic force microscopy topography image of as-prepared WS_2_ QDs. The height profile along the line overlaid on the image is shown in the inset. (color online)

X-ray diffraction (XRD) was employed to further examine the crystal structures of WS_2_ QDs and CD/WS_2_ QDs. The XRD patterns obtained are displayed in Fig. 2a, the diffraction peaks at 2θ = 28.9°, 32°, 33.9°, and 38.0° correspond to (004), (100), (101), and (103) lattice planes of the hexagonal phase WS_2_, respectively. The XRD pattern of the nanocomposite shows that the intrinsic structure of 2H WS_2_ was well retained during the synthesis reaction. For these prepared QD samples, the (002) diffraction peak was not resolved. A few studies have reported similar disappearance or strong suppression of the characteristic (002) diffraction peak for monolayer TMD nanosheets and quantum dots [51–53]. Furthermore, the reflections marked by asterisks were ascribed to the l-cysteine compound [54, 55]. Finally, the thickness of the as-synthesized WS_2_ QDs was checked by atomic force microscopy (AFM) analysis. The AFM height profile shown in Fig. 2b reveals the particle thickness ranging from 6 to 10 nm, which indicates the presence of few-layered QD structure and is close to the TEM results.

### Surface Elemental and Valence State Analysis

**Fig. 3:**
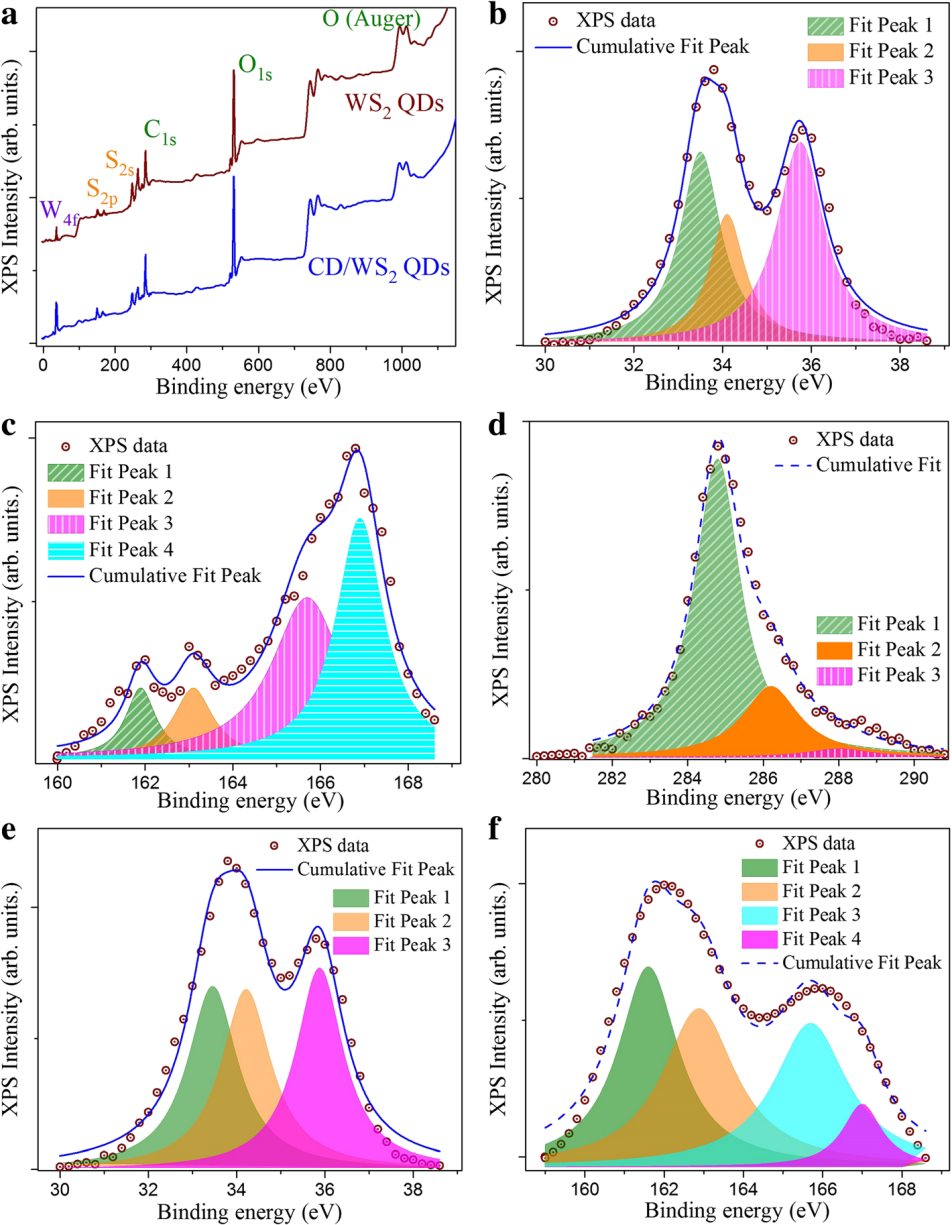
**a** Survey scan spectra of WS_2_ QDs and CD/WS_2_ QD composite. High-resolution XPS spectra showing the binding energies of **b** W 4f, **c** S 2p, **d** C 1s electrons recorded on CD/WS_2_ QD nanocomposite. Core level spectra of **e** W 3d and **f** S 2p recorded on WS_2_ QDs. (color online)

In order to determine the chemical composition and valence states of the elements in the pristine WS_2_ and the CD/WS_2_ QDs, X-ray photoelectron spectroscopy (XPS) analysis was carried out. Figure 3a shows the whole XPS survey spectra of WS_2_ QDs and the CD/WS_2_ QDs. Here, the presence of W, S, C, and O was detected for our synthesized QDs. In the high-resolution W 4f core level spectrum of CD/WS_2_ QDs, the main peak can be deconvoluted to two contributed bands at 33.5 eV and 34.1 eV, as shown in Fig. 3b. They can be assigned to W 4f_7/2_ and W 4f_5/2_ states, and thus confirms the presence of W^4+^ in CD/WS_2_ QDs [41, 56]. One more peak located at 35.7 eV can be assigned to W 5p_3/2_. This can be attributed to the W^6+^ species in the samples [32, 57]. As for the high-resolution S 2p core level spectrum in Fig. 3c, four characteristic peaks with binding energies at 161.9, 163.1, 165.7, and 166.9 eV can be resolved. The S 2p peaks at 161.9 eV and 163.1 eV correspond to S 2p_3/2_ and S2p_1/2_ orbitals of divalent sulfide ions [37, 58]. Together with the binding energy split of 1.2 eV, it indicates the S^2−^ oxidation state in QDs [11, 37]. Meanwhile, the binding energy at 165.7 eV suggests the existence of bridging disulfides S_2_^2−^ and/or apical S^2−^ ligands, which may be related to active edge sites [43, 59]. As for the high-energy component at 166.9 eV, it can be ascribed to S^4+^ species in sulfate groups (SO_3_^2−^), which could locate at edges of WS_2_ QDs [59]. High-resolution spectrum of C 1s is displayed in Fig. 3d. A multiple-peak analysis showed three peaks. The main binding energy peak at 284.7 eV is ascribed to C-C bond, which is due to the carbon atom in graphitic structures. The secondary peak at 286.2 eV is assigned to C-O and/or C-N. Additionally, a minute contribution located at 288.0 eV suggests the presence of C=O bond. The existence of these C 1s peaks is very close to what has been reported for C-dots in the literature [46]. In the case of pristine WS_2_ QDs, analogous XPS spectra shapes were obtained. Figure 3e illustrates the high-resolution W 4f spectrum. It consists of three bands centered at 33.5, 34.2, and 35.8 eV that correspond to the W 4f_7/2_, W 4f_5/2_, and W 5p_3/2_ orbitals, which is reminiscent of hybrid CD/WS_2_ QDs. From Fig. 3f, the fitted peak positions of the detected S 2p spectrum also nearly coincide with the binding energies for pristine WS_2_ QDs. The similarity here hints that the hybridization was mainly realized by a physical adsorption of CDs onto the WS_2_ QD surface instead of formation of covalent bond between the constituting components [30]. The overall XPS results agree with those reported for 2H-WS_2_ and indicate the successful synthesis of WS_2_ QDs [32, 41].

### Optical Property Studies

**Fig. 4:**
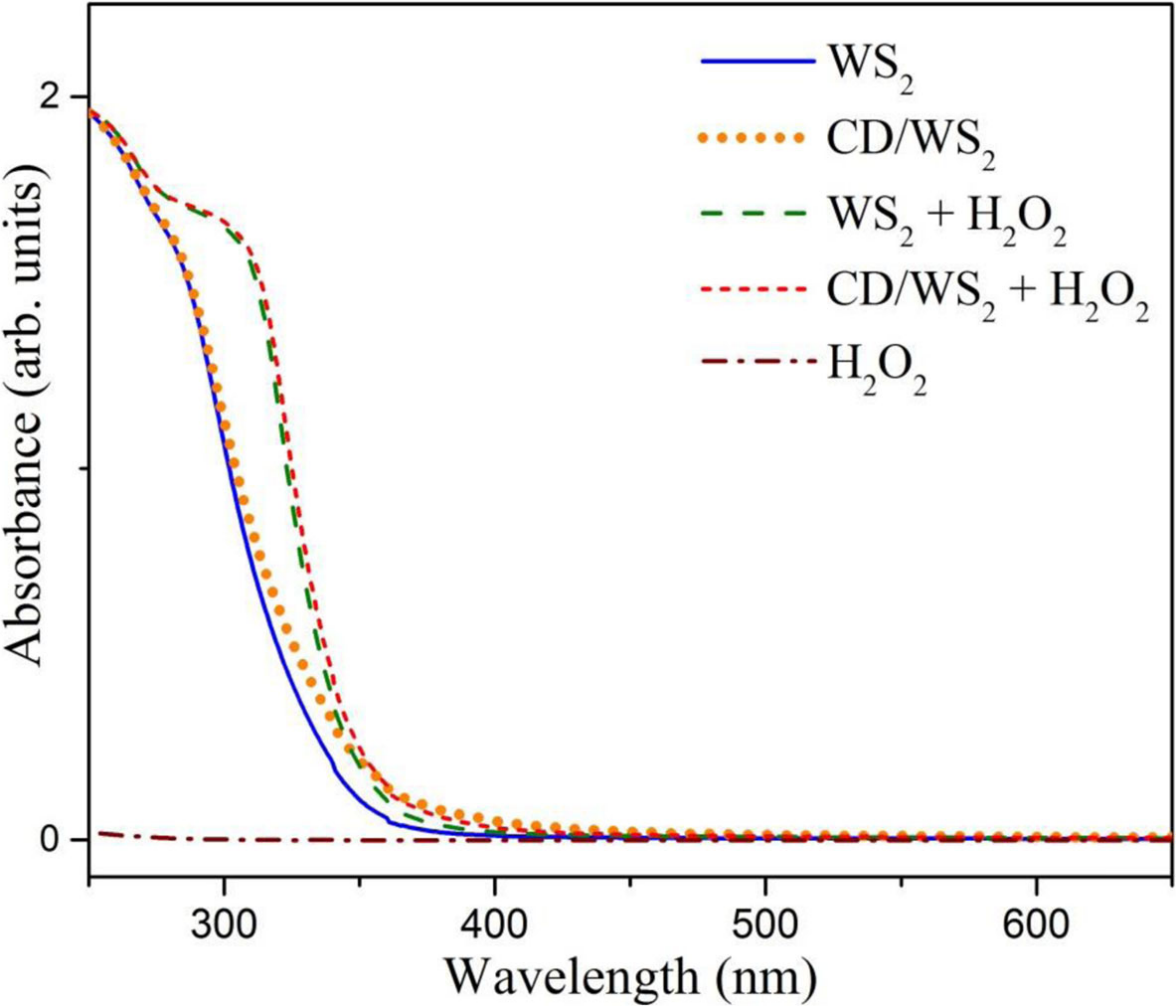
UV–Vis absorption spectra of WS_2_ QDs (blue line) and CD/WS_2_ QDs (orange dot line). UV–Vis spectra of WS_2_ QDs and CD/WS_2_ QDs in the presence of H_2_O_2_ are plotted as green dashed line and red dashed line, respectively. The brown dashed dot line shows the absorbance for H_2_O_2_ alone. (color online)

Optical features of WS_2_ QDs were studied by optical absorption and photoluminescence (PL) measurements. The UV–Vis spectra of our WS_2_ QDs were depicted in Fig. 4. In general, appearance of four characteristic excitonic absorption bands in visible range is expected for WS_2_ microcrystals and 2D nanosheets. Here, the excitonic peaks disappear and dominant absorption bands in the near-UV region (λ ≈ 300 nm) can be observed for as-prepared QDs. The strong absorption is assigned to transitions from the low-lying valence band to the conduction band in WS_2_ QDs. The band-edge position is close to 360 nm, which is due to the quantum size effect. It is known that the optical absorption of TMD QDs exhibits a strong blue-shift when the lateral dimensions of the nanoparticles are less than around 20 nm [50]. As the majority of our fabricated QD sizes are within the quantum confinement regime, a large blue-shift is expected and confirmed.

**Fig. 5:**
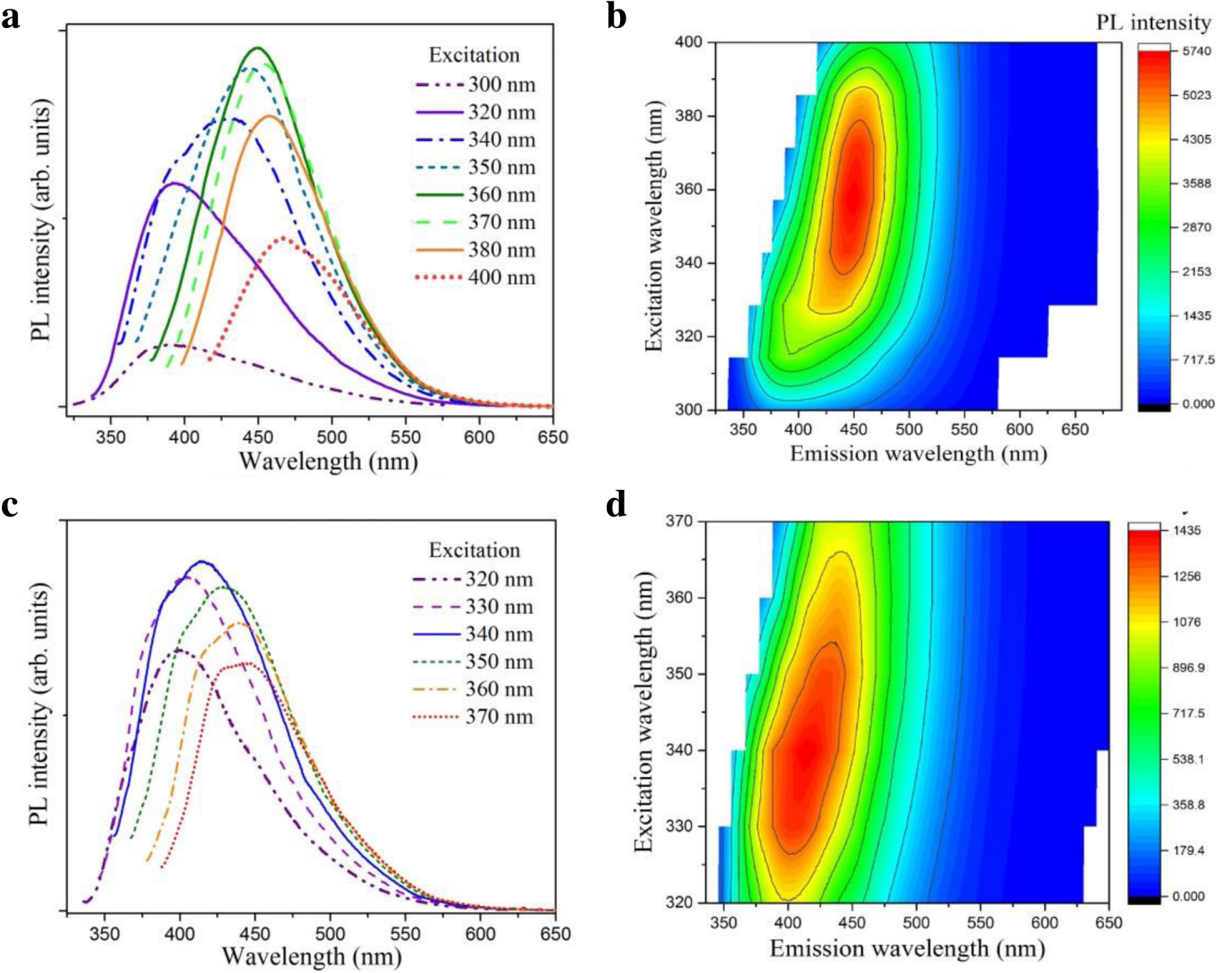
**a** Excitation wavelength-dependent PL spectra of colloidal CD/WS_2_ QDs at room temperature. The peak shift can be attributed to the pronounced QCE. **b** The 2D contour map acquired from the PL spectra. **c** Excitation-dependent PL emission behavior of pristine WS_2_ QDs at room temperature. **d** The 2D color-converted PL intensity map acquired from the spectra. (color online)

**Fig. 6:**
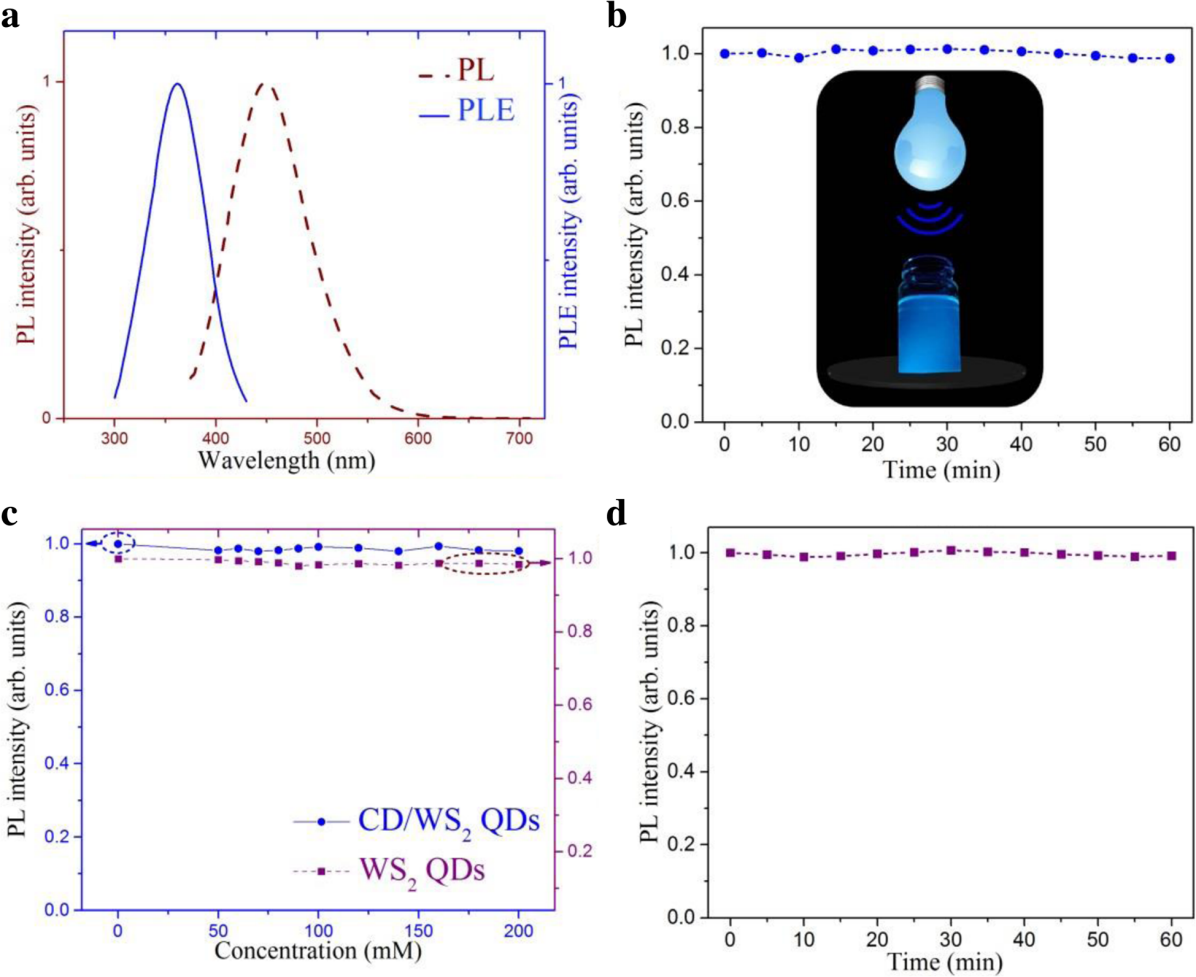
**a** Excitation and emission PL spectra of CD/WS_2_ quantum dots. **b** PL intensity fluctuation of CD/WS_2_ QDs under continuous exposure to 360 nm UV light for 1 h. **c** The ionic stability of PL intensity for CD/WS_2_ QDs (blue circle) and pristine WS_2_ QDs (purple square) with varied NaCl concentrations in the range of 50–200 mM. **d** The temporal stability of PL intensity for pristine WS_2_ QDs for 1 h. (color online)

PL spectroscopy provides a contactless optical means to investigate the electronic structure of semiconductor materials. The PL spectra of the synthesized CD/WS_2_ QD dispersions were taken at room temperature under different excitation wavelengths, as shown in Fig. 5a. As the excitation wavelength was switched from 300 to 400 nm, the emission peak is gradually redshifted from 385 to 470 nm. Analogous excitation-dependent fluorescence emissions have been found in a few TMD QD reports [22, 60]. As found in our UV–Vis results, the QCE strongly affects the band gap of our QDs. A longer wavelength resonantly excites larger QDs with narrower band gaps, leading to emissions peaked at longer wavelengths. Accordingly, the emission peak progressively redshift as the excitation wavelength is increased as a result of the QCE. This trend of PL intensity in response to varied excitation energy is clearly revealed by the 2D color-converted PL contour map as depicted in Fig. 5b. The strongest emission appears at 450 nm (2.58 eV) with an excitation wavelength of 360 nm. The emission may be attributed to excitonic transitions between the minimum of conduction band to the uppermost split valence bands (A and B excitons) [22]. To have deeper insight into the nature of the electronic transitions, PL excitation (PLE) was carried out by using the detection wavelength set at characteristic emission position. Figure 6a displays the PLE spectrum under the detection wavelength of 450 nm. We found an evident PLE peak around 360 nm, which agrees well with the UV–Vis result. It further hints that the strong emission originated from excitonic A emission of QDs [22].

Under irradiation of UV light, a strong blue luminescence can be easily observed by the naked eye, as depicted in the inset of Fig. 6b. It is known that WS_2_ in its bulk form has very limited luminescent intensity. The strong blue emission again supports the successful fabrication of nanostructures in quantum confinement regime. The stability of luminescence is essential in the optical sensing application. The photo stability of CD/WS_2_ QDs was checked by the time-dependent PL measurement under an excitation of 360 nm. Figure 6b shows that the luminescent intensity is almost unchanged after UV irradiation for 1 h. Next, we study the effect of salt solution on the fluorescence intensity of QDs. As presented in Fig. 6c, the CD/WS_2_ QDs possess good ionic stability under different concentrations of NaCl solution, revealing the potential for sensing in a physiological environment. These results suggest that the PL properties of our synthetic QDs can be employed for luminescence sensing purpose. Parallel PL properties were found for pristine WS_2_ QDs except the luminescent intensity is weaker than that of hybrid QDs. Excitation wavelength-dependent PL spectra of pristine WS_2_ QDs are shown in Fig. 5c. Figure 5d displays the 2D PL contour map derived from the PL spectra of WS_2_ QDs, which shows a prominent red-shift with an increase in the excitation wavelength. Good ionic and temporal stability in luminescent intensity was also found for pristine WS_2_ QDs, which is shown in Fig. 6c, d, respectively. The PL quantum yields of WS_2_ QDs and CD/WS_2_ QDs are 3.05% and 4.1% using quinine sulfate as a reference at the excitation wavelength of 360 nm (theoretical quantum yield 54%).

### Application to H_2_O_2_ and Glucose Detection

**Fig. 7:**
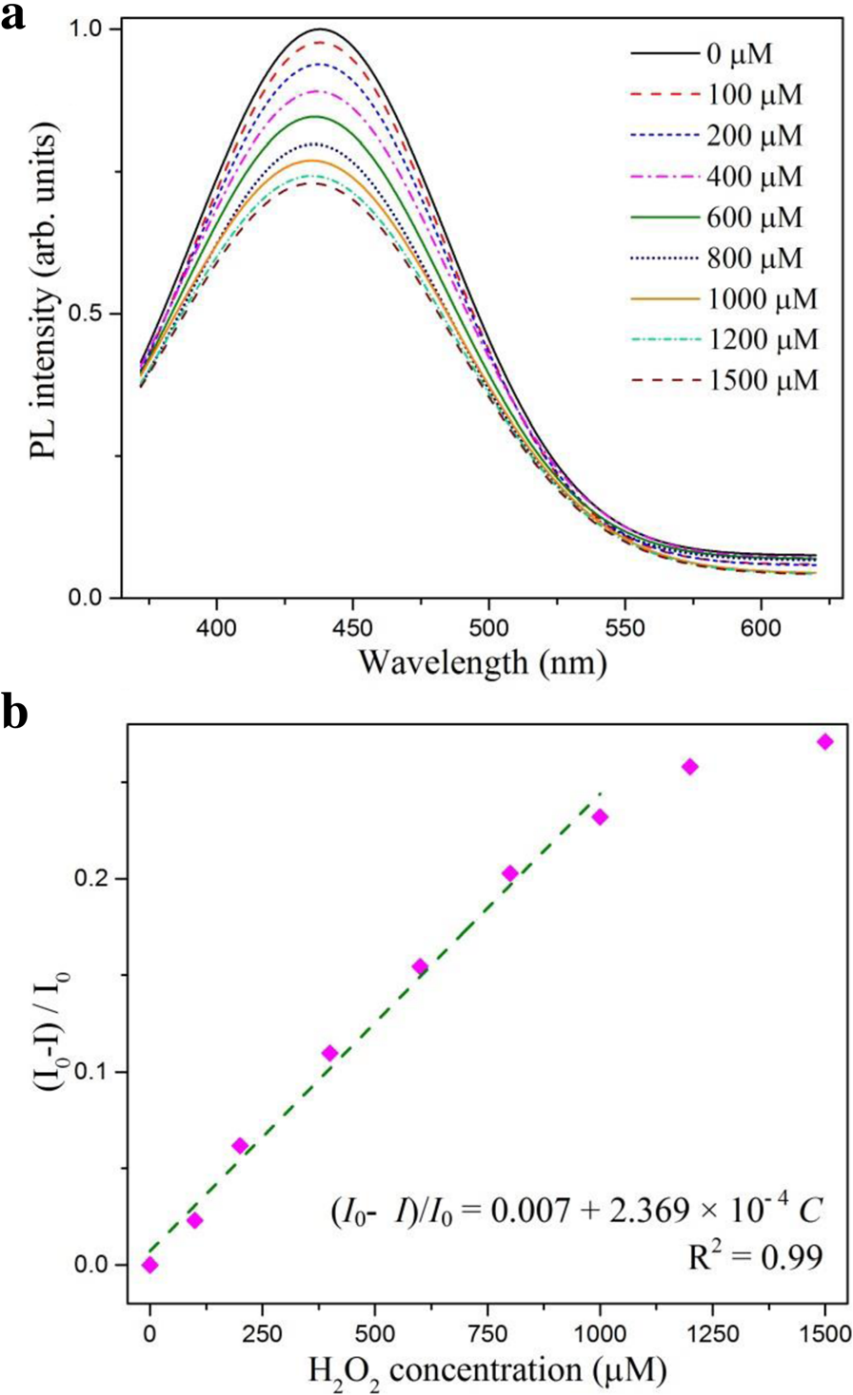
**a** The PL spectra of CD/WS_2_ QDs under 360 nm irradiation with different concentrations of H_2_O_2_. **b** The linear relationship between PL intensity and H_2_O_2_ concentration. (color online)

Different concentrations of H_2_O_2_ were added into both types of WS_2_ QD solutions to evaluate the capability of prepared QDs for luminescence sensing. Figure 7a shows that the PL intensity of the CD/WS_2_ QDs monotonically decreased with increasing the concentration of H_2_O_2_ from 0.1 to 1 mM. The relationship between the H_2_O_2_ concentration and PL intensity is depicted in Fig. 7b. We found the dependence can be fitted as a linear function as (*I*_0_ − *I*)/*I*_0_ = 0.007 + 2.369 × 10^−4^ *C* with a correlation coefficient of *R*^2^ = 0.99, where *I*_0_ and *I* were the PL intensity of sensing system in the absence and presence of target molecules, respectively. The detection limit is estimated to be 40 μM. For pristine WS_2_ QDs, the PL spectra with varied concentrations of H_2_O_2_ are shown in Additional file 1: Figure S1 (a). A good linear relationship was also obtained in the same concentration range with *R*^2^ = 0.99 and a detection limit of 60 μM was assessed, as presented in Additional file 1: Figure S1 (b). The linear detection range is quite similar to a recent H_2_O_2_ optical sensing study on the use MoS_2_ QDs [24].

**Fig. 8:**
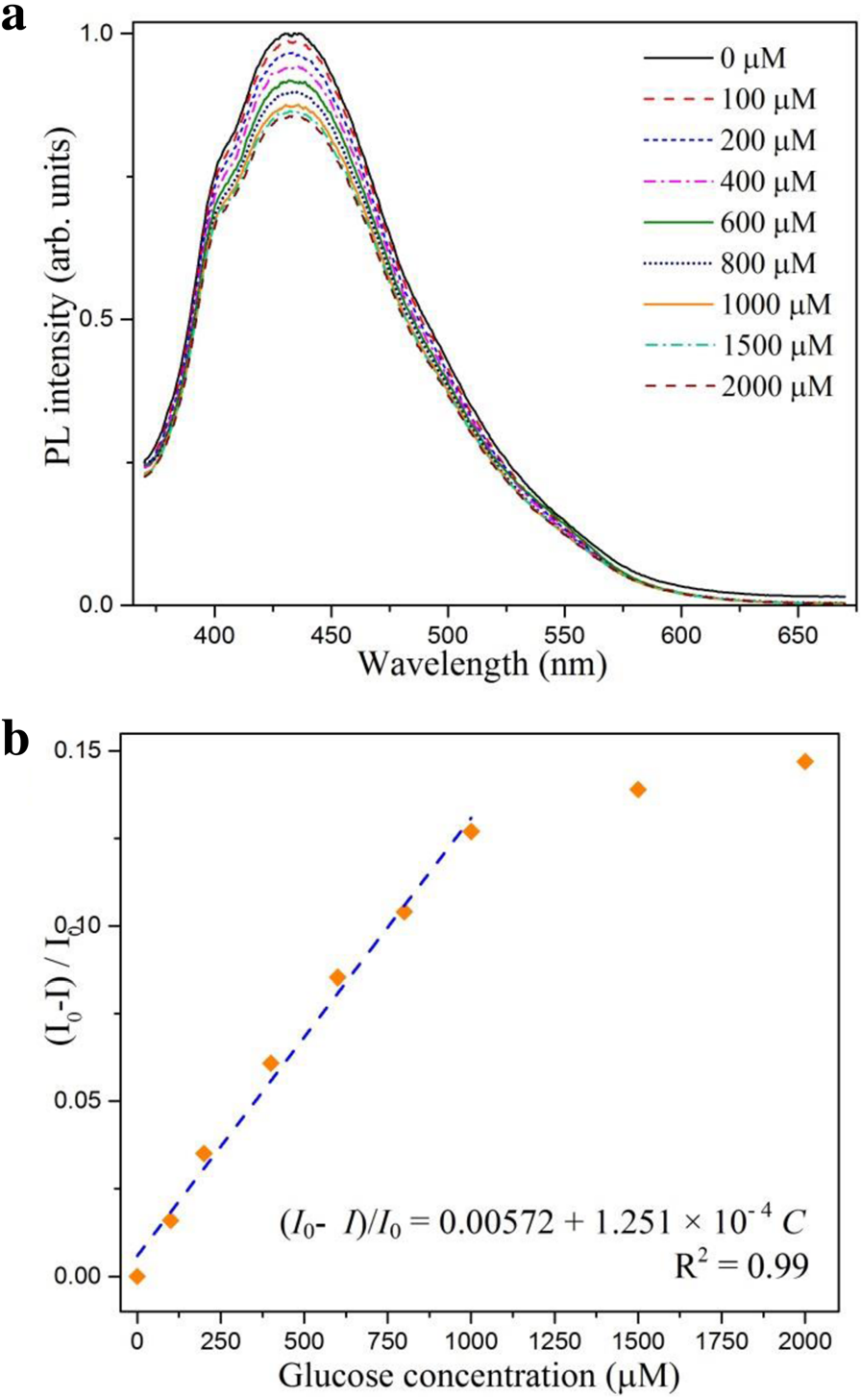
**a** The PL spectra of CD/WS_2_ QDs under 360 nm irradiation with different amounts of glucose. **b** The corresponding linear calibration plot for glucose sensing. (color online)

The developed fluorescence sensing system was further extended to the measurement of glucose. In the presence of glucose oxidase (GOx) in solution, glucose can be oxidized to gluconic acid with dissolved oxygen, as illustrated in Scheme 1. The main reaction product H_2_O_2_ can then trigger the PL quenching of WS_2_ QDs in proportion, which serves as the basis for glucose detection. The PL intensity of the CD/WS_2_ QDs with different amount of glucose is shown in Fig. 8a. In the company of GOx, the PL intensity decreased progressively with the increase of the concentration of glucose from 0.1 to 1 mM, which is due to the increasing amount of produced H_2_O_2_. Figure 8b exhibits a good linear relationship between the quenching efficiency and glucose concentration (*R*^2^ = 0.99 and LOD = 60 μM). As for pristine WS_2_ QDs, the glucose concentration-dependent PL spectra are displayed in Additional file 1: Figure S2 (a). There exists a good linear relationship in the concentration range of 0.8 to 8 mM, as shown in Additional file 1: Figure S2 (b). This LOD is larger than that of CD/WS_2_ QDs. Our result shows that CD/WS_2_ QDs provide a more sensitive LOD for glucose detection while pristine WS_2_ QDs works better for larger dynamic range.

**Fig. 9:**
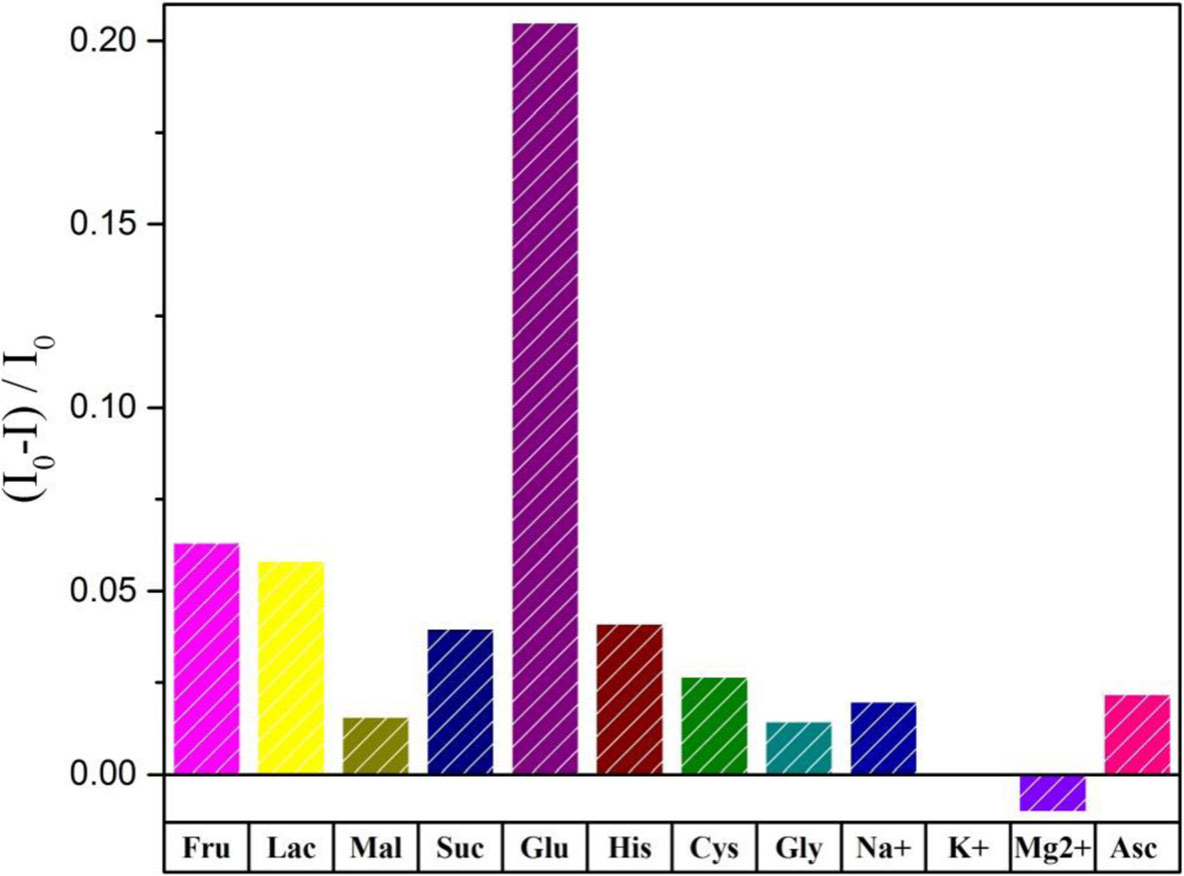
Selectivity tests for glucose detection using other sugars and some usual species as control. (color online)

To further assess the selectivity of this glucose sensing platform, control experiments were carried out to compare the quenching efficiency induced by fructose, lactose, maltose, and some other species. As illustrated in Fig. 9, these glucose analogs caused little impact on glucose detection, which is due to the high affinity of GOx. Meanwhile, the others posed insignificant changes in the probe signals. Therefore, our results suggest that WS_2_ QDs can be employed as an alternative platform for the optical determination of glucose level.

### Time-Resolved PL and Raman Studies

**Fig. 10:**
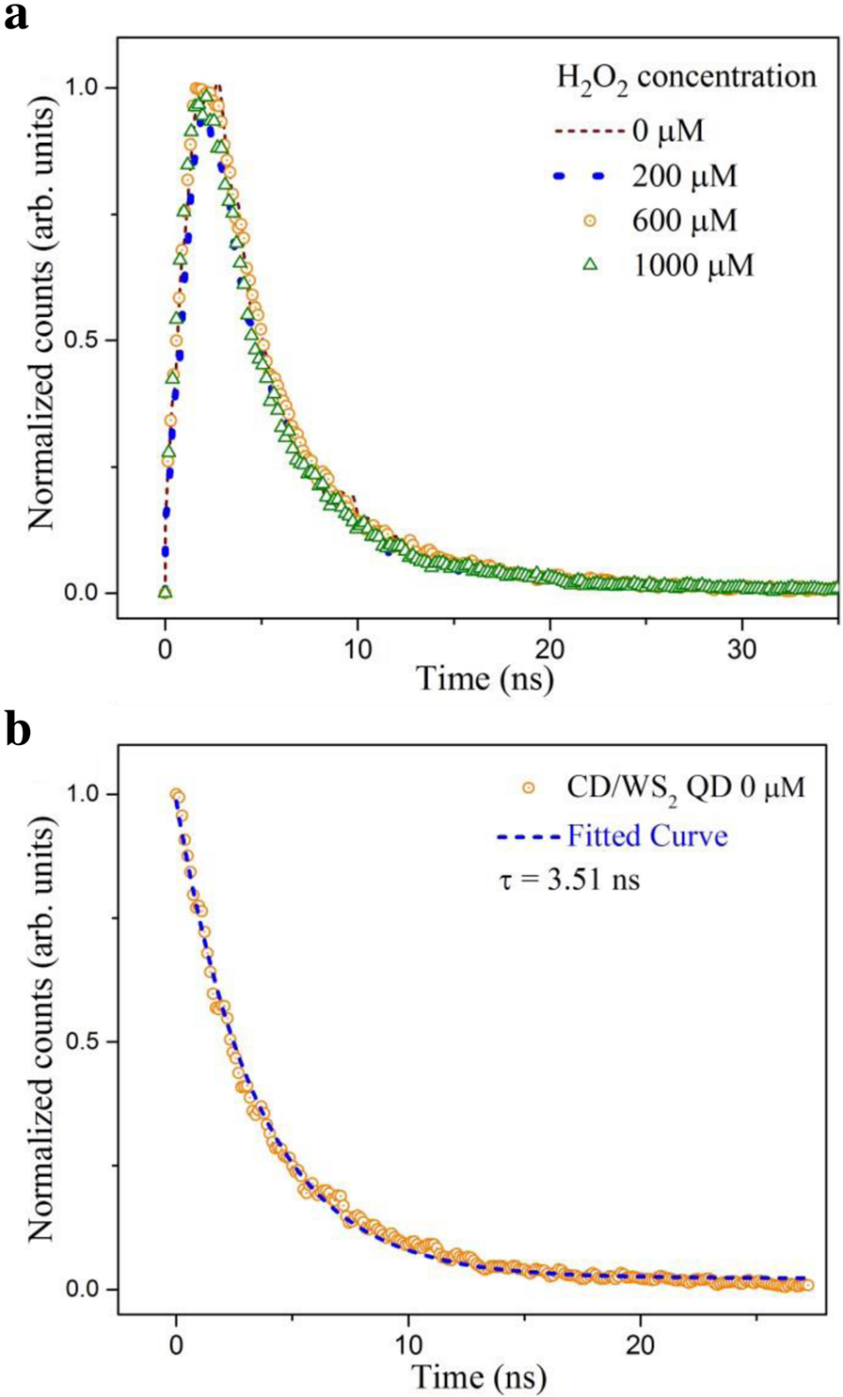
**a** Time-resolved PL spectra of CD/WS_2_ QDs with and without the presence of hydrogen peroxide. **b** PL decay curve in the absence of hydrogen peroxide (orange dots). The dashed line represents the fit to the experimental data. (color online)

To further explore the photo physical properties of the fluorescence system, more optical investigations were imposed. Time-resolved PL (TRPL) was recorded at the strongest emission wavelength ≈ 450 nm by using an excitation wavelength of 360 nm. The TRPL spectrum of CD/WS_2_ QD solutions was depicted as the brown dashed line in Fig. 10a. The decay behavior indicates a nanosecond-scale lifetime of luminescence. Its decay kinetics can be fitted well with a single exponential decay function, as plotted in Fig. 10b. The lifetime of luminescence was estimated to be 3.51 ns. Moreover, we found that when the QD solutions were treated with different concentrations of H_2_O_2_, no eloquent changes could be observed to the PL decay curves. Calculated lifetimes of TRPL spectra were summarized in Additional file 1: Table S1. Identical properties were also observed for pristine WS_2_ QDs, as shown in Additional file 1: Figure S3. Our results indicate that the recombination dynamics in QDs are barely affected by hydrogen peroxide so that the lifetime of photo-generated excitons is almost unchanged. As a consequence, the suppression of PL cannot be ascribed to a reduction in transition rate or an increase in nonradiative traps [61].

**Fig. 11:**
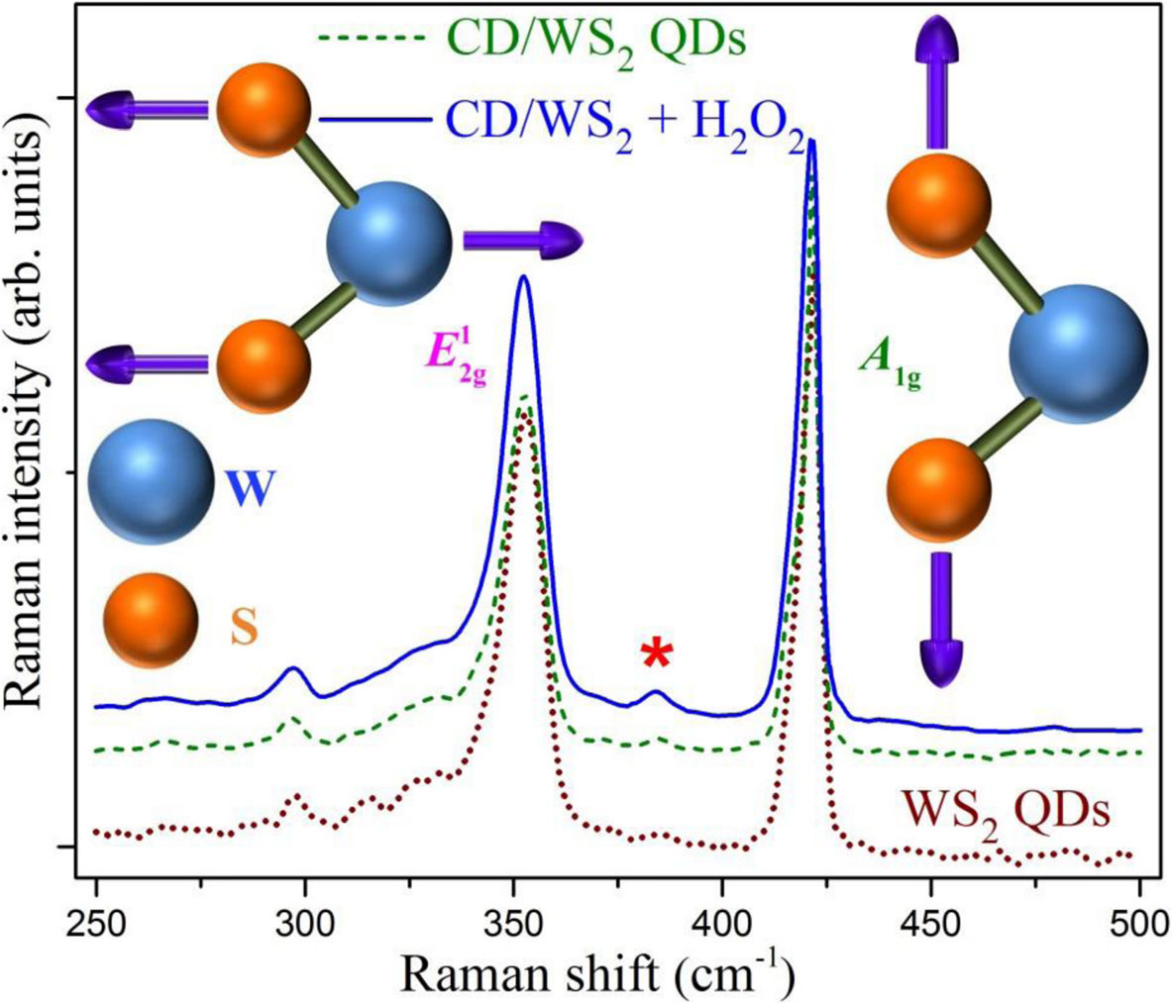
Raman spectra of WS_2_ QDs and CD/WS_2_ QDs. The Raman spectrum of CD/WS_2_ QDs after hydrogen peroxide treatment is shown as the solid line. The inset sketch illustrates the atomic displacements for the two vibrational modes responsible for the primary Raman peaks. (color online)

Raman spectroscopy has been frequently employed to extract additional complementary information of ultrathin 2D-layered nanomaterials [62]. In general, for 2D-layered TMD compounds, there are four Raman-active modes, specifically A_1g_, E_1g_, E^1^_2g_, and E^2^_2g_ modes [62, 63]. E_1g_ mode is hardly found in 2D nanosheet reports because of forbidden selection rule in the typical back-scattering measurement geometry. The representative Raman spectra of pristine WS_2_ and CD/WS_2_ QDs were displayed in Fig. 11. Two major peaks at 353 cm^−1^ and 420 cm^−1^ reveal the clear signature of WS_2_ in all the prepared samples. The inset sketch illustrates the two principal Raman-active modes of WS_2_, which lead to the two peaks in the Raman spectra. The A_1g_ mode at 420 cm^−1^ results from the out-of-plane vibration of S atoms in opposite direction. Besides, we observed small shoulder on the lower-frequency side of the A_1g_ peak, which arises due to Davydov splitting as reported earlier [64, 65]. Due to the lattice stiffening effect of the A_1g_ mode, the Raman shift between the main A_1g_ and the in-plane E^1^_2g_ modes has been employed as an indicator of WS_2_ thickness [66, 67]. Here, the energy splitting between the two peaks are almost identical and the frequency difference of 67 cm^−1^ suggests the few-layer structure of our WS_2_-based QDs [67]. Another proposed gauge of sample thickness is the ratio of the intensity of A_1g_ mode to that of E^1^_2g_ mode. The A_1g_ peak is 1.35 and 1.6 times the height of the E^1^_2g_ peak for WS_2_ and CD/WS_2_ QDs, respectively. It also reveals the few-layer nature of our synthesized QD structures [67]. Notably, the slightly larger Raman peak ratio of CD/WS_2_ QDs reflects the increased physical thickness of WS_2_ QDs in the hybridization process. The common weak feature at 297 cm^−1^ is close to the E_1g_ mode whose appearance could be related to 2D few-layer QD structure [68, 69]. Similar feature found by other group has been proposed to be a multi-phonon scattering mode [70]. Here, both modes may coexist in our Raman observation [69].

One other interesting characteristic was noted in the Raman scattering results of CD/WS_2_ QDs after H_2_O_2_ treatment. As designated by an asterisk in Fig. 11, there exists an identifiable signal at 385 cm^−1^, which is attributable to neither first-order nor second-order WS_2_ Raman scattering modes [68]. This peak can be ascribed to the bending (δ) mode O–W–O in WS_2_ QDs [71, 72], whose presence indicates the formation of W–O bonds upon H_2_O_2_ treatment. This mode became obviously pronounced because of the oxidation induced by hydrogen peroxide. As edge states are abundant in ultrathin 2D QDs, partial oxidation or doping of oxygen is facilitated in the reactions with hydrogen peroxide. It is in sharp contrast with 2D nanosheets because sheet surfaces are not very sensitive to oxidation. Recently, a first-principles calculation showed that the band structure of partially oxidized MoS_2_ QDs can be modified, leading to the suppression of photoluminescence by hydrogen peroxide treatment [61]. It was shown that with certain degree of oxidation, the high efficient direct bandgap structure of MoS_2_ QDs can become inefficient indirect bandgap structure with certain bandgap narrowing. In this case, the photoluminescence of oxidized MoS_2_ QDs can be quenched and additional longer wavelength absorption could be found. These effects predicted by the above-mentioned calculations are consistent with our experimental outcome in partially oxidized WS_2_ QDs. Analogous mechanism is very likely to occur in our case since general features of the WS_2_ band structure are similar to those of MoS_2_. Furthermore, we found the corresponding absorption band of two types of WS_2_ QDs appeared red-shift after H_2_O_2_ was added to the solution, as shown by the dashed lines in Fig. 4. As a comparison, the absorption data of sole hydrogen peroxide was included as the brown dashed dot line, which indicates that the change is not due to the presence of H_2_O_2_ alone. Same behavior was recently reported for oxidation-induced luminescence quenching of MoS_2_ QDs [24]. Consequently, oxidation induced by hydrogen peroxide is accounted for the sensing mechanism of our WS_2_ QDs by using PL quenching.

## Conclusions

In summary, for the first time, photoluminescent WS_2_ QDs and CD/WS_2_ QDs were prepared under “bottom-up” hydrothermal conditions by using sodium tungstate dihydrate and l-cysteine. From the TEM analysis, it can be observed that the synthesized WS_2_ QDs had high crystallinity and featured good dispersibility. On the basis of the strong PL with high stability from as-prepared QDs, they were subsequently applied for the construction of an electrodeless PL quenching sensor for detection of H_2_O_2_ and glucose. Both types of QDs show similar capability in H_2_O_2_ sensing and hybrid CD/WS_2_ QDs provide a more sensitive LOD for glucose detection. The stability test showed that the produced WS_2_-based QDs are robust against photo-degradation and is stable during the sensing period. The Raman study implied that H_2_O_2_ causes the partial oxidation of QDs, which may lead to oxidation-induced quenching. Compared with most reported works with “top-down” approaches, the proposed “bottom-up” protocol for WS_2_-based QDs has the advantages of simple preparation, low cost, eco-friendliness, and ease for hybrid construction. Furthermore, these water-soluble WS_2_-based QDs with abundant active sites can be a promising candidate for potential applications in environmental monitoring, biochemistry, and clinical diagnostics. For instance, as there exist numerous kinds of O_2_-dependent oxidases which generates hydrogen peroxide, the presented facile 0D QDs may also be employed to detect other target molecules by taking the corresponding enzymes. Overall, our results provide an alternative and cost-efficient platform to exploit the diverse functionalities of 0D WS_2_-based nanomaterials. Further structural layout and extended applications are underway.

## Additional file

Additional file 1: **Figure S1.** (a) PL spectra of WS2 QDs under 360 nm irradiation with different concentrations of H2O2. (b) The linear calibration plot for H2O2 concentration. **Figure S2.** (a) The PL spectra of WS_2_ QDs under 360 nm irradiation with different amounts of glucose. (b) The correlation between PL quenching ratios and the concentration of glucose. **Figure S3.** Time-resolved PL spectra of WS_2_ QDs treated with an increasing concentration of hydrogen peroxide. **Table S1.** Calculated lifetime of TRPL spectra of CD/WS_2_ QDs treated with varied concentration of hydrogen peroxide. (DOCX 404 kb)

## Data Availability

All data generated or analyzed during this study are included in this published article and its supplementary information file.

## Abbreviations

AFM: Atomic force microscopy
CD: Carbon quantum dot
GOx: Glucose oxidase
HRTEM: High-resolution transmission electron microscopy
PL: Photoluminescence
PLE: Photoluminescence excitation
QCE: Quantum confinement effect
QDs: Quantum dots
TEM: Transmission electron microscopy
TMD: Transition metal dichalcogenide
TRPL: Time-resolved photoluminescence
UV–Vis: Ultraviolet-visible
XPS: X-ray photoelectron spectroscopy
XRD: X-ray diffractometer
