# Supplementary material for: Facile Bottom-up Preparation of WS2-Based Water-Soluble Quantum Dots as Luminescent Probes for Hydrogen Peroxide and Glucose
Source: Nanoscale Res Lett. 2019 Aug 9;14:271. doi: 10.1186/s11671-019-3109-5 (PMC6689045; doi:10.1186/s11671-019-3109-5)
Supplement: Supplementary file 1 — Figure S1. (a) PL spectra of WS2 QDs under 360 nm irradiation with different concentrations of H2O2. (b) The linear calibration plot for H2O2 concentration. Figure S2. (a) The PL spectra of WS2 QDs under 360 nm irradiation with different amounts of glucose. (b) The correlation between PL quenching ratios and the concentration of glucose. Figure S3. Time-resolved PL spectra of WS2 QDs treated with an increasing concentration of hydrogen peroxide. Table S1. Calculated lifetime of TRPL spectra of CD/WS2 QDs treated with varied concentration of hydrogen peroxide. (DOCX 404 kb) [file 11671_2019_3109_MOESM1_ESM.docx]

**Supporting information**

**Facile bottom-up preparation of WS_2_-based water-soluble quantum dots as luminescent probes for hydrogen peroxide and glucose**

**Da-Ren Hang^a,b*^, De-You Sun^a^, Chun-Hu Chen^c^, Hui-Fen Wu^c^, Mitch M. C. Chou^a,b^, Sk Emdadul Islam^a^, and Krishna Hari Sharma^a^**

*^a^Department of Materials and Optoelectronic Science, National Sun Yat-sen University, Kaohsiung 80424, Taiwan*

*^b^Center of Crystal Research, National Sun Yat-sen University, Kaohsiung 80424, Taiwan*

*^b^Department of Chemistry, National Sun Yat-sen University, Kaohsiung 80424, Taiwan*

^*^ Electronic mail: drhang@faculty.nsysu.edu.tw


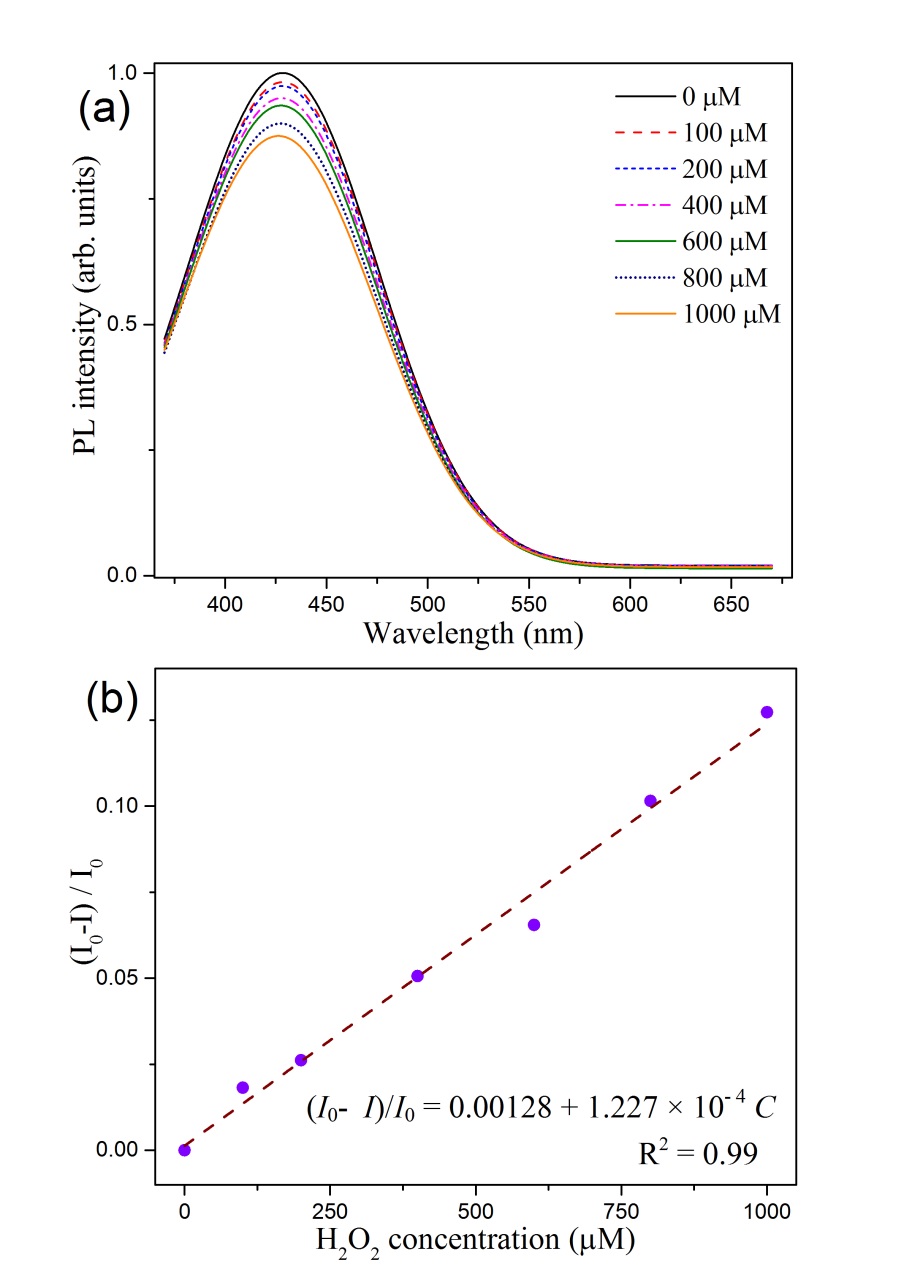


**Figure S1**. (a) PL spectra of WS_2_ QDs under 360 nm irradiation with different concentrations of H_2_O_2_. (b) The linear calibration plot for H_2_O_2_ concentration.


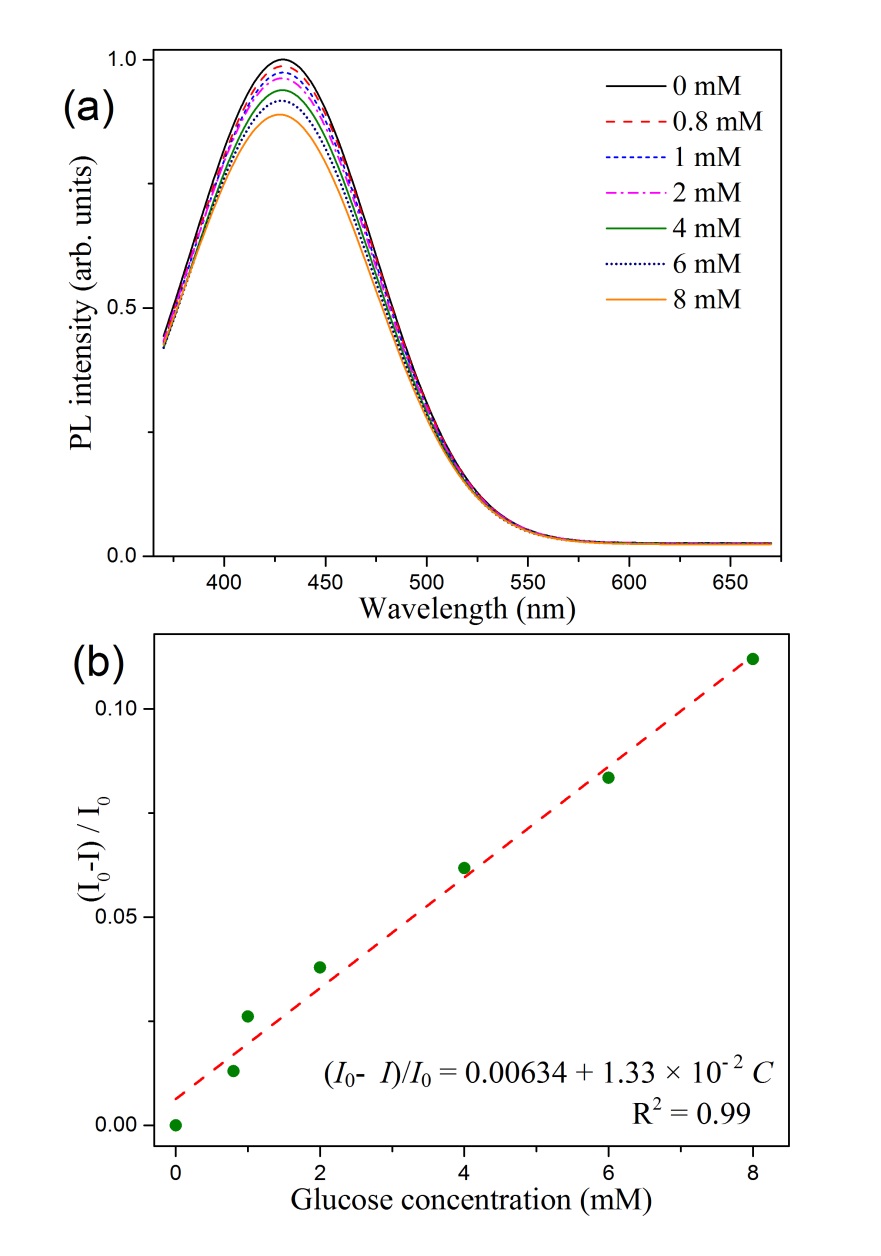


**Figure S2**. (a) The PL spectra of WS_2_ QDs under 360 nm irradiation with different amounts of glucose. (b) The correlation between PL quenching ratios and the concentration of glucose.


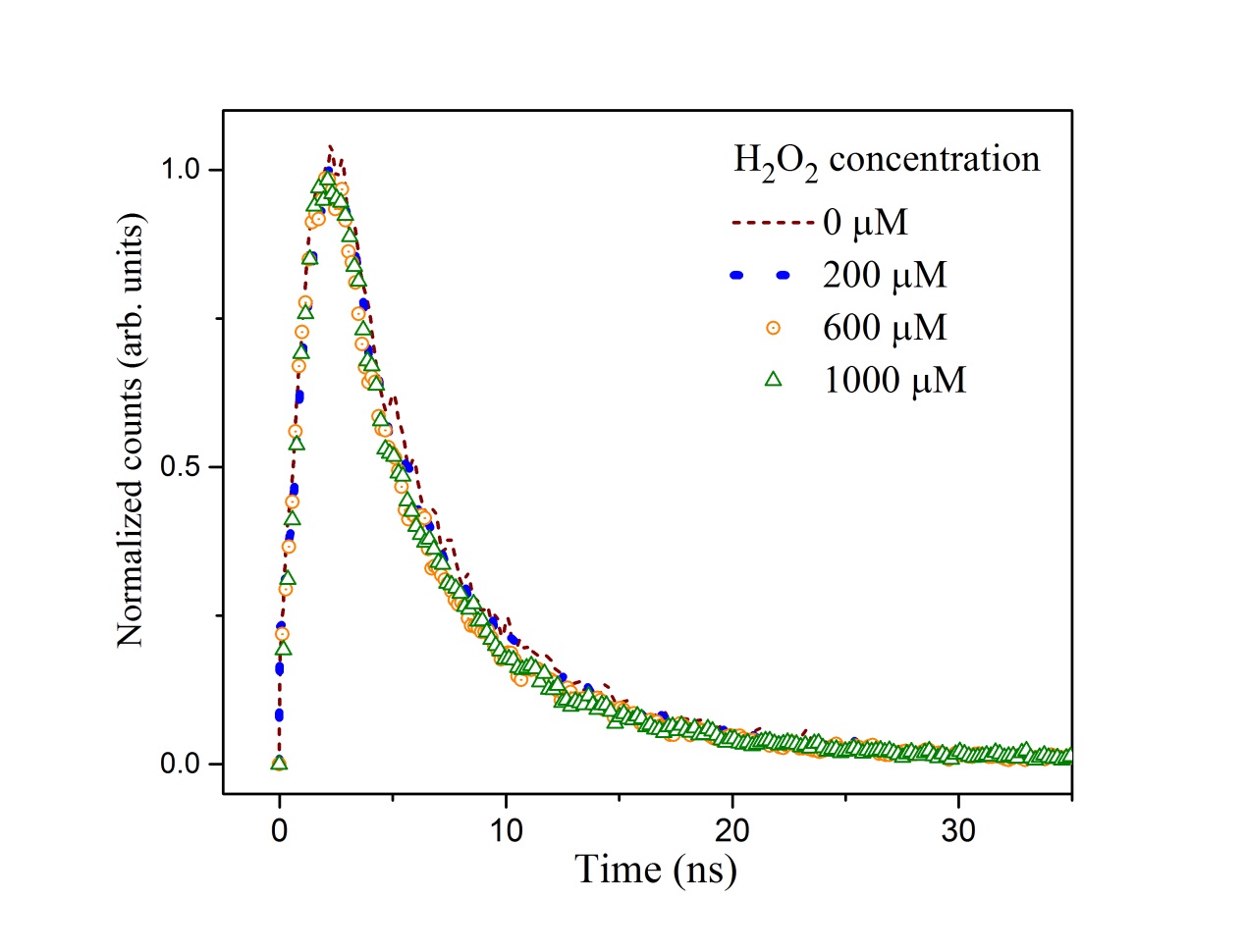


**Figure S3**. Time-resolved PL spectra of WS_2_ QDs treated with an increasing concentration of hydrogen peroxide.

| H_2_O_2_ concentration (μM) | 0 | 200 | 600 | 1000 |
| --- | --- | --- | --- | --- |
| Lifetime | 3.51 | 3.62 | 3.71 | 3.52 |

**Table S1**. Calculated lifetime of TRPL spectra of CD/WS_2_ QDs treated with varied concentration of hydrogen peroxide.
